# *catena*-Poly[[lithium-μ_2_-(di­hydrogen pyrazine-2,3,5,6-tetra­carboxyl­ato)-κ^6^ *O* ^2^,*N* ^1^,*O* ^6^;*O* ^3^,*N* ^4^,*O* ^5^-lithium-di-μ-aqua-κ^4^ *O*:*O*] 2.5-hydrate]

**DOI:** 10.1107/S160053681401174X

**Published:** 2014-05-24

**Authors:** Wojciech Starosta, Janusz Leciejewicz

**Affiliations:** a Institute of Nuclear Chemistry and Technology, ul.Dorodna 16, 03-195 Warszawa, Poland

## Abstract

The title coordination polymer, {[Li_2_(C_8_H_2_N_2_O_8_)(H_2_O)_2_]·2.5H_2_O}_*n*_, is built up from mol­ecular ribbons propagating in the *c*-axis direction of the ortho­rhom­bic unit cell; the ligand bridges two Li^+^ ions using both its *N*,*O*,*O*′-bonding sites and adjacent Li^+^ ions are bridged by pairs of water mol­ecules. The coordination geometry of the metal ion is distorted trigonal bipyramidal, with the ligand O atoms in the axial sites. Two of the carboxyl­ate groups of the ligand remain protonated and form short symmetric O—H⋯O hydrogen bonds. In the crystal, the ribbons inter­act *via* a network of O—H⋯O hydrogen bonds in which coordinating water mol­ecules act as donors and carboxyl­ate O atoms within adjacent ribbons act as acceptors, giving rise to a three-dimensional framework. O—H⋯N inter­actions are also observed. The asymmetric unit contains quarter of the ligand and the complete ligand has 2/*m* symmetry; the Li^+^ ion lies on a special position with *m*.. site symmetry. Both bridging water mol­ecules have *m*2*m* site symmetry and both lattice water mol­ecules have *m*.. site symmetry; one of the latter was modelled with a site occupancy of 0.25.

## Related literature   

For the structures of related lithium complexes with pyrazine-2,3,5,6-tetra­carboxyl­ate and water ligands, see: Starosta & Leciejewicz (2010 ▶, 2014 ▶). 
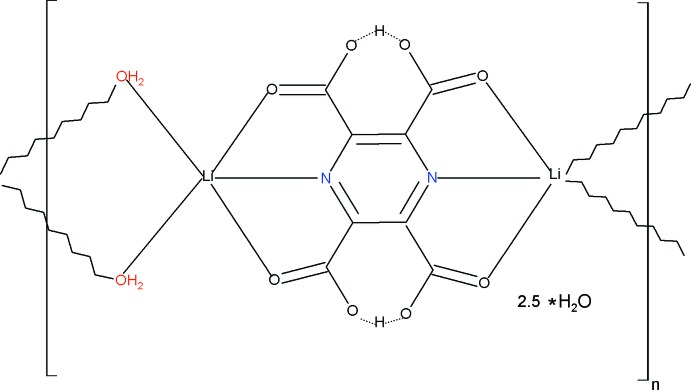


## Experimental   

### 

#### Crystal data  

[Li_2_(C_8_H_2_N_2_O_8_)(H_2_O)_2_]·2.5H_2_O
*M* *_r_* = 174.53
Orthorhombic, 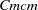

*a* = 12.0554 (3) Å
*b* = 6.39040 (17) Å
*c* = 19.3383 (4) Å
*V* = 1489.80 (6) Å^3^
*Z* = 8
Mo *K*α radiation
μ = 0.15 mm^−1^
*T* = 567 K
0.24 × 0.20 × 0.05 mm

#### Data collection  

Agilent SuperNova (Dual, Cu at zero, Eos) diffractometer
Absorption correction: multi-scan (*CrysAlis PRO*; Agilent, 2011 ▶) *T*_min_ = 0.701, *T*_max_ = 1.000
7496 measured reflections
1151 independent reflections
997 reflections with *I* > 2σ(*I*)
*R*_int_ = 0.029

#### Refinement  

*R*[*F* ^2^ > 2σ(*F* ^2^)] = 0.042
*wR*(*F* ^2^) = 0.122
*S* = 1.07
1151 reflections
85 parameters
3 restraints
H atoms treated by a mixture of independent and constrained refinement
Δρ_max_ = 0.25 e Å^−3^
Δρ_min_ = −0.29 e Å^−3^

### 

Data collection: *CrysAlis PRO* (Agilent, 2011 ▶); cell refinement: *CrysAlis PRO*; data reduction: *CrysAlis PRO*; program(s) used to solve structure: *SHELXS97* (Sheldrick, 2008 ▶); program(s) used to refine structure: *SHELXL97* (Sheldrick, 2008 ▶); molecular graphics: *SHELXTL* (Sheldrick, 2008 ▶); software used to prepare material for publication: *SHELXTL*.

## Supplementary Material

Crystal structure: contains datablock(s) I, New_Global_Publ_Block. DOI: 10.1107/S160053681401174X/hb7224sup1.cif

Structure factors: contains datablock(s) I. DOI: 10.1107/S160053681401174X/hb7224Isup2.hkl

CCDC reference: 1004410

Additional supporting information:  crystallographic information; 3D view; checkCIF report

## Figures and Tables

**Table 1:** Selected bond lengths (Å)

Li1—O1	2.1538 (13)
Li1—O3	1.982 (4)
Li1—O4	1.976 (4)
Li1—N1	2.116 (3)

**Table 2:** Hydrogen-bond geometry (Å, °)

*D*—H⋯*A*	*D*—H	H⋯*A*	*D*⋯*A*	*D*—H⋯*A*
O6—H62⋯N1	0.87 (2)	2.28 (11)	2.886 (12)	127 (12)
O5—H5⋯O1^i^	0.85 (2)	1.95 (2)	2.7711 (13)	163 (2)
O3—H3⋯O5^ii^	0.82 (4)	1.92 (4)	2.730 (2)	173 (4)
O4—H4⋯O5^iii^	0.90 (3)	1.83 (3)	2.726 (2)	179 (3)
O2—H2⋯O2^iv^	1.21 (1)	1.21 (1)	2.409 (3)	175 (1)

Symmetry codes: (i) 
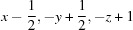
; (ii) 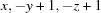; (iii) 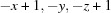; (iv) 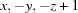.

**Fig. 1.**
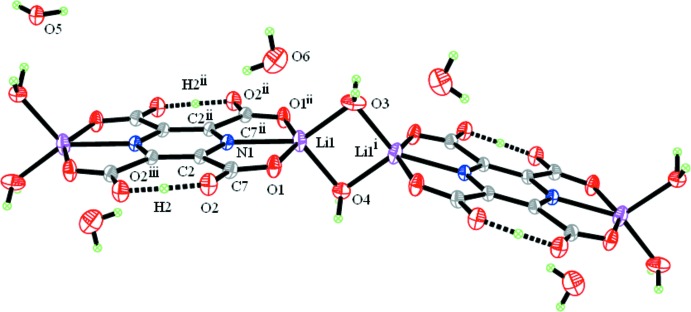
A fragment of a [001] chain in the title compound with 50% probability displacement ellipsoids. Symmetry code: ix, y, -z + 1; ii -x + 1, y, z; iiix, -y, -z + 1.

**Fig. 2.**
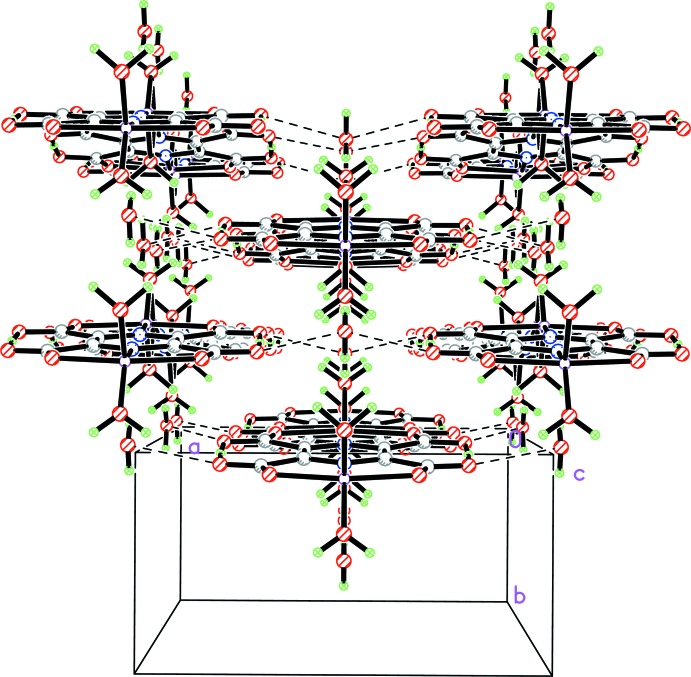
The alignment of the ribbons viewed along the crystal a direction.

## supplementary crystallographic information

### 1. Comment

The structure of the title compound is composed of molecular ribbons propagating in the crystal [001] direction. The structural unit of a ribbon is built of a centro-symmetric ligand molecule which bridges two Li ions using its both *N,O,O* bonding sites. Two water molecules chelated simultaneously to Li ions belonging to adjacent structural units, bridge them into a molecular ribbon [Fig.1]. The coordination environment of a Li ion is distorted trigonal bipyramidal. Its equatorial plane is composed of coplanar Li1, N1,O3,O4 atoms; O1 and O1^i^ are at the apices. The Li—O and Li—N bond distances are usual (Table 2). Within a ligand two carboxylate groups remain protonated to maintain charge balance and form short, intramolecular symmetric hydrogen bonds of 2.409 (1) Å (Table 3). The ligand ring is almost planar (r.m.s. is 0.0003 (1) Å; the carboxylate C17/O1/O12 group makes with it a dihedral angle of 2.6 (1)°. Bond distances and bond angles within the hetero-ring do not differ from those reported in the structures of two other Li complexes with the title ligand (Starosta & Leciejewicz, 2010, 2014). The Fourier map shows two solvation water molecules (O5 and O6), both at special positions. The refinemt reveals a disorder of the O6 aqua molecule with 0.25 positional occupancy *i.e.* two molecules at random in a unit cell. This molecule locates coplanarly with the N1, O3, O4 and Li1 atoms at a distance of 2.538 (2) Å from the latter. The ribbons are held together by a system of hydrogen bonds in which coordinated and solvation water molecules act as donors and carboxylate O atoms as acceptors giving rise to a three-dimensional framework. The structures of two other Li complexes with the title ligand have been recently reported. The structural unit of the title polymer is closely related to that one observed in the structure of the first compound which cosists of discrete dimeric molecules built of two aqua-coordinated Li ions bridged by the ligand molecule *via* both its N,*O*,*O* bonding sites (Starosta & Leciejewicz, 2014). The structure of the second complex is built of anions each consisting of an aqua coordinated Li ion cheleted to a doubly deprotonated ligand molecule and of aqua coordinated Li cations (Starosta & Leciejewicz, 2010).

### 2. Experimental

An aqueous solution containing 2 mmol of lithium nitrate and a small excess over 1 mmol of pyrazine-2,3,5,6-tetracarboxylic acid dihydrate was heated under reflux with stirring at *ca* 330 K for 10 h. After cooling to room temperature the solution was left to evaporate. Three days later well formed colorless blocks of the title compound were found, which were washed with cold methanol and dried in the air.

### 3. Refinement

Water and carcoxylate H atoms were found in the Fourier map and refined isotropically.

### Crystal data

**Table:**

[Li(C_8_H_2_N_2_O_8_)(H_2_O)_2_]·2.5H_2_O	*F*(000) = 716
*M**_r_* = 349.06	*D*_x_ = 1.556 Mg m^−^^3^
Orthorhombic, *C**m**c**m*	Mo *K*α radiation, λ = 0.71073 Å
Hall symbol: -C 2c 2	Cell parameters from 3296 reflections
*a* = 12.0554 (3) Å	θ = 3.8–31.9°
*b* = 6.39040 (17) Å	µ = 0.15 mm^−^^1^
*c* = 19.3383 (4) Å	*T* = 293 K
*V* = 1489.80 (6) Å^3^	Block, colourless
*Z* = 4	0.24 × 0.20 × 0.05 mm

### Data collection

**Table:**

Agilent SuperNova (Dual, Cu at zero, Eos) diffractometer	1151 independent reflections
Radiation source: SuperNova (Mo) X-ray Source	997 reflections with *I* > 2σ(*I*)
Mirror monochromator	*R*_int_ = 0.029
Detector resolution: 16.0131 pixels mm^-1^	θ_max_ = 30.0°, θ_min_ = 3.4°
ω scans	*h* = −16→16
Absorption correction: multi-scan (*CrysAlis PRO*; Agilent, 2011)	*k* = −8→8
*T*_min_ = 0.701, *T*_max_ = 1.000	*l* = −27→27
7496 measured reflections	

### Refinement

**Table:**

Refinement on *F*^2^	Primary atom site location: structure-invariant direct methods
Least-squares matrix: full	Secondary atom site location: difference Fourier map
*R*[*F*^2^ > 2σ(*F*^2^)] = 0.042	Hydrogen site location: inferred from neighbouring sites
*wR*(*F*^2^) = 0.122	H atoms treated by a mixture of independent and constrained refinement
*S* = 1.07	*w* = 1/[σ^2^(*F*_o_^2^) + (0.0592*P*)^2^ + 0.9014*P*] where *P* = (*F*_o_^2^ + 2*F*_c_^2^)/3
1151 reflections	(Δ/σ)_max_ < 0.001
85 parameters	Δρ_max_ = 0.25 e Å^−^^3^
3 restraints	Δρ_min_ = −0.29 e Å^−^^3^

### Special details

**Table:**

Geometry. All e.s.d.'s (except the e.s.d. in the dihedral angle between two l.s. planes) are estimated using the full covariance matrix. The cell e.s.d.'s are taken into account individually in the estimation of e.s.d.'s in distances, angles and torsion angles; correlations between e.s.d.'s in cell parameters are only used when they are defined by crystal symmetry. An approximate (isotropic) treatment of cell e.s.d.'s is used for estimating e.s.d.'s involving l.s. planes.
Refinement. Refinement of *F*^2^ against ALL reflections. The weighted *R*-factor *wR* and goodness of fit *S* are based on *F*^2^, conventional *R*-factors *R* are based on *F*, with *F* set to zero for negative *F*^2^. The threshold expression of *F*^2^ > σ(*F*^2^) is used only for calculating *R*-factors(gt) *etc*. and is not relevant to the choice of reflections for refinement. *R*-factors based on *F*^2^ are statistically about twice as large as those based on *F*, and *R*- factors based on ALL data will be even larger.

### Fractional atomic coordinates and isotropic or equivalent isotropic displacement parameters (Å^2^)

**Table:**

	*x*	*y*	*z*	*U*_iso_*/*U*_eq_	Occ. (<1)
O1	0.67161 (8)	0.07053 (19)	0.35355 (5)	0.0435 (3)	
O4	0.5000	−0.1345 (3)	0.2500	0.0388 (4)	
O2	0.79263 (8)	0.04117 (18)	0.43921 (5)	0.0413 (3)	
O5	0.33035 (11)	0.4166 (2)	0.7500	0.0379 (3)	
O3	0.5000	0.3004 (4)	0.2500	0.0533 (6)	
N1	0.5000	0.0321 (2)	0.43101 (7)	0.0270 (3)	
C2	0.59630 (8)	0.01679 (18)	0.46407 (6)	0.0259 (3)	
C7	0.69421 (10)	0.0441 (2)	0.41465 (6)	0.0310 (3)	
Li1	0.5000	0.0823 (7)	0.32284 (17)	0.0463 (8)	
H4	0.555 (2)	−0.229 (5)	0.2500	0.070 (9)*	
H3	0.445 (3)	0.377 (7)	0.2500	0.097 (13)*	
H5	0.2920 (19)	0.432 (3)	0.7135 (10)	0.068 (7)*	
H2	0.797 (3)	0.0000	0.5000	0.097 (12)*	
O6	0.5000	0.4486 (17)	0.3733 (5)	0.077 (2)	0.25
H61	0.5000	0.578 (7)	0.386 (7)	0.115*	0.25
H62	0.5000	0.39 (2)	0.413 (4)	0.115*	0.25

### Atomic displacement parameters (Å^2^)

**Table:**

	*U*^11^	*U*^22^	*U*^33^	*U*^12^	*U*^13^	*U*^23^
O1	0.0302 (5)	0.0732 (8)	0.0270 (5)	−0.0022 (4)	0.0051 (3)	0.0037 (4)
O4	0.0299 (9)	0.0406 (10)	0.0459 (11)	0.000	0.000	0.000
O2	0.0207 (4)	0.0659 (7)	0.0374 (5)	−0.0024 (4)	0.0029 (3)	0.0018 (4)
O5	0.0240 (6)	0.0562 (9)	0.0334 (7)	0.0024 (6)	0.000	0.000
O3	0.0336 (11)	0.0356 (11)	0.0908 (18)	0.000	0.000	0.000
N1	0.0207 (6)	0.0370 (7)	0.0232 (6)	0.000	0.000	−0.0010 (5)
C2	0.0198 (5)	0.0335 (6)	0.0244 (5)	−0.0005 (4)	0.0010 (4)	−0.0025 (4)
C7	0.0226 (5)	0.0410 (7)	0.0295 (6)	−0.0015 (4)	0.0048 (4)	−0.0021 (5)
Li1	0.0421 (18)	0.070 (2)	0.0271 (15)	0.000	0.000	0.0075 (15)
O6	0.049 (4)	0.101 (7)	0.081 (5)	0.000	0.000	−0.020 (5)

### Geometric parameters (Å, º)

**Table:**

O1—C7	1.2242 (16)	O3—Li1^ii^	1.982 (4)
Li1—O1	2.1538 (13)	O3—H3	0.82 (4)
Li1—O1^i^	2.1538 (13)	N1—C2	1.3289 (12)
Li1—O3	1.982 (4)	N1—C2^i^	1.3289 (12)
Li1—O4	1.976 (4)	C2—C2^iii^	1.406 (2)
Li1—N1	2.116 (3)	C2—C7	1.5287 (15)
O4—Li1^ii^	1.976 (4)	Li1—O6	2.536 (11)
O4—H4	0.90 (3)	Li1—Li1^ii^	2.817 (7)
O2—C7	1.2780 (15)	O6—H61	0.86 (2)
O2—H2	1.2057 (19)	O6—H62	0.87 (2)
O5—H5	0.85 (2)		
			
C7—O1—Li1	119.00 (12)	O3—Li1—O1	102.74 (10)
Li1^ii^—O4—Li1	91.0 (2)	N1—Li1—O1	73.87 (9)
Li1^ii^—O4—H4	118.2 (11)	O4—Li1—O1^i^	99.91 (12)
Li1—O4—H4	118.2 (11)	O3—Li1—O1^i^	102.74 (10)
C7—O2—H2	113.8 (18)	N1—Li1—O1^i^	73.87 (9)
Li1—O3—Li1^ii^	90.6 (3)	O1—Li1—O1^i^	147.72 (17)
Li1—O3—H3	114.8 (18)	O4—Li1—O6	157.2 (3)
Li1^ii^—O3—H3	114.8 (18)	O3—Li1—O6	67.9 (3)
C2—N1—C2^i^	121.76 (13)	N1—Li1—O6	76.1 (3)
C2—N1—Li1	119.12 (7)	O1—Li1—O6	85.76 (13)
C2^i^—N1—Li1	119.12 (7)	O1^i^—Li1—O6	85.76 (13)
N1—C2—C2^iii^	119.12 (7)	O4—Li1—Li1^ii^	44.52 (11)
N1—C2—C7	111.44 (10)	O3—Li1—Li1^ii^	44.70 (13)
C2^iii^—C2—C7	129.43 (6)	N1—Li1—Li1^ii^	171.28 (12)
O1—C7—O2	124.56 (11)	O1—Li1—Li1^ii^	106.01 (9)
O1—C7—C2	116.56 (11)	O1^i^—Li1—Li1^ii^	106.01 (9)
O2—C7—C2	118.87 (11)	O6—Li1—Li1^ii^	112.6 (3)
O4—Li1—O3	89.22 (15)	Li1—O6—H61	174 (10)
O4—Li1—N1	126.8 (2)	Li1—O6—H62	85 (10)
O3—Li1—N1	144.0 (2)	H61—O6—H62	101 (3)
O4—Li1—O1	99.91 (12)		
			
C2^i^—N1—C2—C2^iii^	0.1 (3)	Li1^ii^—O3—Li1—O6	180.000 (2)
Li1—N1—C2—C2^iii^	−179.94 (18)	C2—N1—Li1—O4	90.02 (13)
C2^i^—N1—C2—C7	−178.85 (10)	C2^i^—N1—Li1—O4	−90.02 (13)
Li1—N1—C2—C7	1.1 (2)	C2—N1—Li1—O3	−89.98 (13)
Li1—O1—C7—O2	−177.66 (16)	C2^i^—N1—Li1—O3	89.98 (13)
Li1—O1—C7—C2	1.3 (2)	C2—N1—Li1—O1	−0.42 (18)
N1—C2—C7—O1	−1.57 (17)	C2^i^—N1—Li1—O1	179.54 (12)
C2^iii^—C2—C7—O1	179.61 (16)	C2—N1—Li1—O1^i^	−179.55 (12)
N1—C2—C7—O2	177.46 (12)	C2^i^—N1—Li1—O1^i^	0.42 (18)
C2^iii^—C2—C7—O2	−1.4 (2)	C2—N1—Li1—O6	−89.98 (13)
Li1^ii^—O4—Li1—O3	0.0	C2^i^—N1—Li1—O6	89.98 (13)
Li1^ii^—O4—Li1—N1	180.0	C2—N1—Li1—Li1^ii^	90.02 (13)
Li1^ii^—O4—Li1—O1	−102.80 (12)	C2^i^—N1—Li1—Li1^ii^	−90.02 (13)
Li1^ii^—O4—Li1—O1^i^	102.80 (12)	C7—O1—Li1—O4	−126.14 (15)
Li1^ii^—O4—Li1—O6	0.000 (4)	C7—O1—Li1—O3	142.40 (14)
Li1^ii^—O3—Li1—O4	0.0	C7—O1—Li1—N1	−0.56 (19)
Li1^ii^—O3—Li1—N1	180.0	C7—O1—Li1—O1^i^	1.0 (5)
Li1^ii^—O3—Li1—O1	99.99 (14)	C7—O1—Li1—O6	76.2 (3)
Li1^ii^—O3—Li1—O1^i^	−99.99 (14)	C7—O1—Li1—Li1^ii^	−171.48 (10)

Symmetry codes: (i) −*x*+1, *y*, *z*; (ii) *x*, *y*, −*z*+1/2; (iii) *x*, −*y*, −*z*+1.

### Hydrogen-bond geometry (Å, º)

**Table:**

*D*—H···*A*	*D*—H	H···*A*	*D*···*A*	*D*—H···*A*
O6—H62···N1	0.87 (2)	2.28 (11)	2.886 (12)	127 (12)
O5—H5···O1^iv^	0.85 (2)	1.95 (2)	2.7711 (13)	163 (2)
O3—H3···O5^v^	0.82 (4)	1.92 (4)	2.730 (2)	173 (4)
O4—H4···O5^vi^	0.90 (3)	1.83 (3)	2.726 (2)	179 (3)
O2—H2···O2^iii^	1.21 (1)	1.21 (1)	2.409 (3)	175 (1)

Symmetry codes: (iii) *x*, −*y*, −*z*+1; (iv) *x*−1/2, −*y*+1/2, −*z*+1; (v) *x*, −*y*+1, −*z*+1; (vi) −*x*+1, −*y*, −*z*+1.
